# Conceptual Approach of Diffusion- and Perfusion-Weighted Magnetic Resonance Imaging in Chest Diseases

**DOI:** 10.5334/jbr-btr.1223

**Published:** 2016-11-19

**Authors:** Johan Coolen

**Affiliations:** 1 Department of Radiology, Universitary Hospitals of Leuven, Leuven, BE

**Keywords:** Diffusion-weighted MR, Dynamic Contrast Enhanced MR, Functional MR, Pleural Diseases, Mediastinal Diseases, Lung Nodule, Tissue Characterization

## Abstract

The present manuscript discusses the development of a quantitative, and ultimately a visual approach as well, for detecting, diagnosing, staging, and following-up chest diseases.

At the moment, computer tomography (CT) and positron emission tomography (PET) are the modalities of choice, and despite repeated attempts to integrate magnetic resonance imaging (MRI) in thoracic imaging diagnosis protocols, the classic sequences have – outside radiation reduction – usually no additional benefit in diagnosis. During this thesis, the attempt was made to show that by means of functional imaging sequences a better characterization of pleural, mediastinal and lung lesions was feasible. We even evaluated early treatment response by using diffusion-weighted imaging (DWI) as biomarker. Where possible, the correlation was made between radiological and histopathological images.

## Body

We first developed a diagnostic imaging protocol with functional magnetic resonance (MR) sequences, in particular diffusion-weighted (DW-) and dynamic contrast-enhanced (DCE-) MR for lesion characterization in pleural, lung and mediastinal diseases. In the second phase we focused on the evaluation of early chemoradiotherapy response and outcome by using DW-MR as a biomarker for patients with inoperable malignant pleural mesothelioma (MPM stage III and IV) and extensive disease small cell lung cancer (SCLC-ED).

In the first study we found a significant difference in apparent diffusion coefficient (ADC, calculated from the DW-MR) between malignant and benign pleural disease. Using a quantitative, purely ADC-based diagnostic approach, the optimal threshold to differentiate benign lesions from malignant pleural disease, in a cohort of 31 patients was 1.52 x 10^–^³ mm²/s, with a sensitivity, specificity and accuracy of 71.4% [10/14], 100% [17/17], and 87.1% [27/31], respectively.

**Figure 1:**
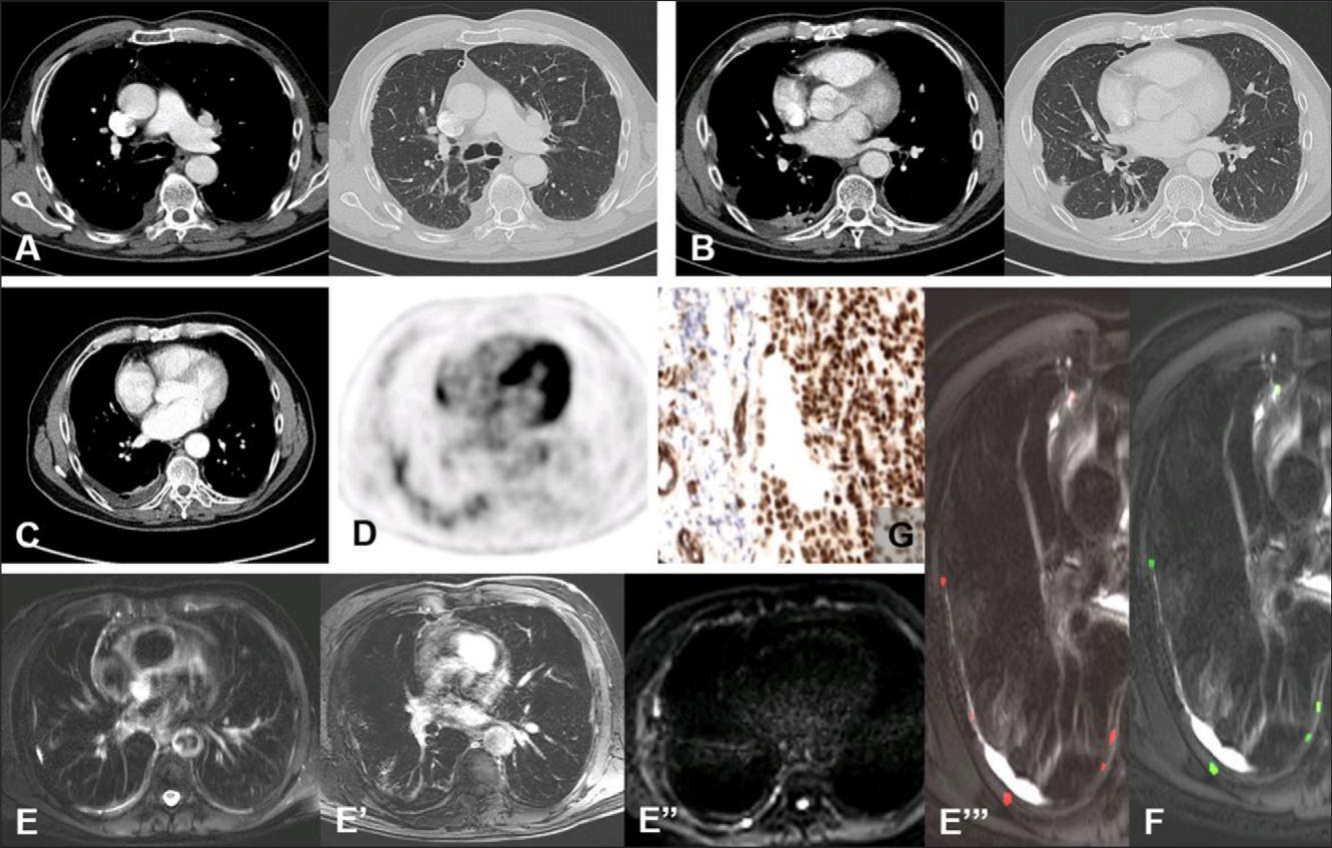
A 50-year-old man with chronic, atypical right chest pain. Pleuroscopy was performed and initial diagnosis of chronic pleurisy due a history of asbestos exposure was made. **(A and B)** CT images before pleurodesis and **(C and D)** PET-CT images after talc pleurodesis showed no additional features. **(E and E’)** The anatomical T2-weighted MR image as well as the T1-weighted MR image after Gadolinium give no additional information. **(E’’)** The EPI-based DWI sequence showed the pointillism sign, **(E’’’)** visible on B1000 overlay on the T2-weighted MR image. **(F)** The MPA folder showed no risk-zones (all MPM foci are green spots) but the presence of a pointillism sign must alert us for possible underlying malignancy. **(G)** The surgical biopsies were positive, TTF-1 stains were positive for epitheloid MPM.

This result could be improved to 92.8% [13/14], 94.1% [16/17] and 93.5% [29/31], respectively, when DCE-MR imaging data were included in those cases were ADC was between 1.52 and 2.00 x 10^–3^ mm²/s [1]. Because quantitative interpretation of functional MR sequences is quite time consuming for clinicians, we compared visual assessments of different imaging parameters for evaluating malignant pleural mesothelioma (MPM). Beside mediastinal pleural thickness (accuracy, 78% [78/100]) and the sign of lung shrinkage (accuracy, 66% [66/100]), we found a better accuracy of 88% [88/100] for pleural pointillism [2]. We introduced this sign to describe the presence of multiple hyperintense spots at high b-value DW-MR imaging, not attributable to T2 shine-through. We agree that a definitive diagnosis of MPM normally requires histologic sampling with Haematoxylin-Eosin staining and often additional histochemical stains. But pleural pointillism has a number of advantages with clinical relevance. This visual sign needs no calculation, it can be mapped easily and appear even before the ADC changes become measurable (Figure 1). Integrated ^18^FDG-PET-CT is now considered the standard technique for staging of MPM with reported sensitivity, specificity and accuracy of 67%, 93% and 83%, respectively [3].

**Figure 2:**
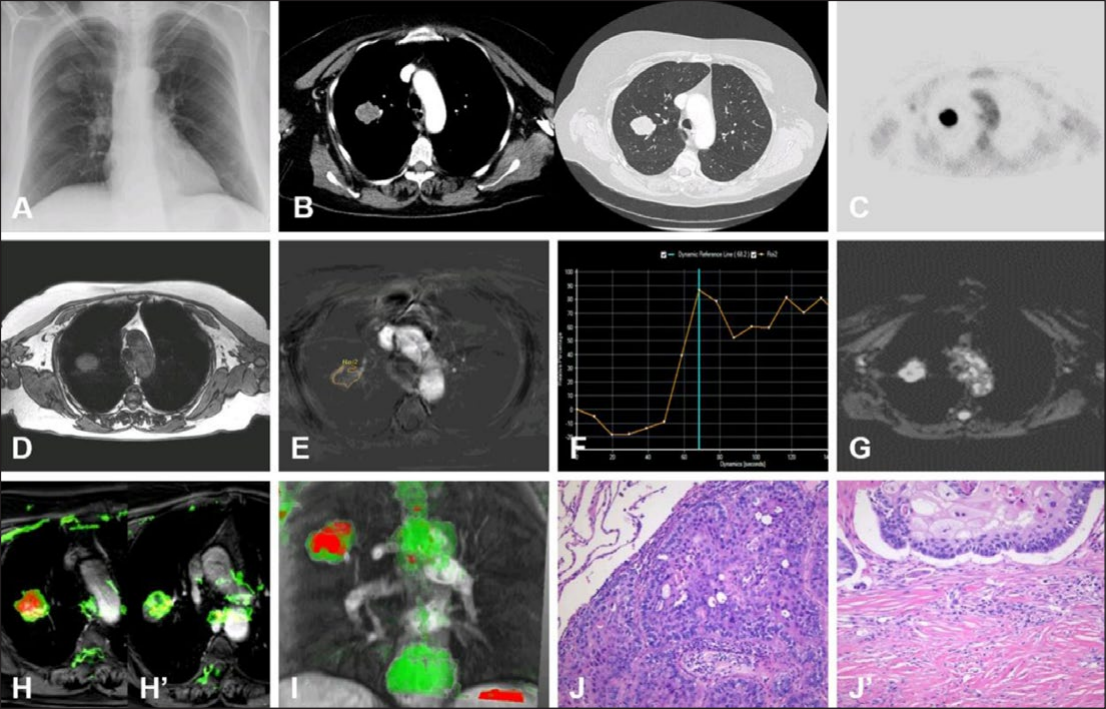
**(A)** CXR of a 65-year-old woman with chronic fatigue and a family history of rheumatism. The right suprahilar region showed a SPL. **(B and C)** PET-CT confirms a FDG-avid nodule in the right upper lobe. An MR examination with **(D)** T2-weighted MR sequence and **(E)** DCE-MR sequence showed **(F)** a type B enhancement curve, with additional **(G)** DWI showing a restrictive lesion in the malignant range (ADCavg 1.11x10^–^³ mm²/s). MPA differences found in the **(H)** cranial and **(H’)** caudal areas of the lesion confirm inhomogeneity of the SPL, with **(I)** malignant tissue cranial and benign foci in the basal pole. Histopathological correlation was made with **(J)** H&E staining, with findings of moderate differentiated squamous cell carcinoma in the upper pole. **(J’)** The basal pole of the specimen consist of discrete fibrosis with smooth muscle cell hyperplasia.

In the second research project we evaluated preoperative single pulmonary lesions (SPL). In the literature sensitivity, specificity and accuracy of 96%, 88% and 93%, respectively are mentioned for SPL diagnosis using integrated PET-CT [4]. However, based on earlier research of Schaefer [5] we decided upon another strategy. In a feasibility group, we found that combining conventional MR sequences with visual interpretation of DCE-MR curves resulted in a sensitivity, specificity and accuracy of 100% [43/43], 55% [6/11] and 91% [49/54], respectively. These results could be improved by DW-MR (with a cut-off value of 1.52 x 10^–^³ mm²/s for ADChigh, calculated from b values>300 sec/mm²) leading to a sensitivity, specificity and accuracy of 98% [42/43], 82% [9/11] and 94% [51/54], respectively. Afterwards, a separate validation group was accrued in which these results were confirmed [6]. Therefore, we could conclude that visual DCE-MR-based curve interpretation can be used for initial differentiation of benign from malignant SPL, while additional quantitative DWI-based interpretation can further improve the specificity. Introduction of a multiparametric approach (MPA) also enables the possibility to access the inhomogeneity of lesions (Figure 2).

In the third study we examined the problem of lesion inhomogeneity on mediastinal lesion characterization and improved the prediction of mediastinal lesion resectability. To tackle the issue of lesion heterogeneity, we evaluated 100 consecutive patients with mediastinal lesions and performed an evaluation in four steps. First we evaluated the whole lesion on the lowest b-value DW-MR image (b = 0 sec/mm²) as well as the areas of the lesions that remained bright at higher b-values (measured b = 1000 and extrapolated b = 2000 sec/mm²). Honing the delineation to only include the b = 2000 hyperintensities improved the lesion differentiation (ADC cut-off of 2.00 x 10^–^³ mm²/sec) leading to a sensitivity, specificity and accuracy of 95% [57/60], 87.5% [35/40] and 92% [92/100], respectively. While these b = 2000 delineations lead to quite good accuracies, false positive findings did occur with more variability due to operator- and scanner-dependent extrapolations. Therefore, we introduced in a fourth step, a more visual and direct innovative *multiparametric analysis* (MPA) approach [7]. This MPA is an approach reducible to clustering of different parametric values (b0, ADC and DCE-MR-based), leading to *target zones*.

**Figure 3:**
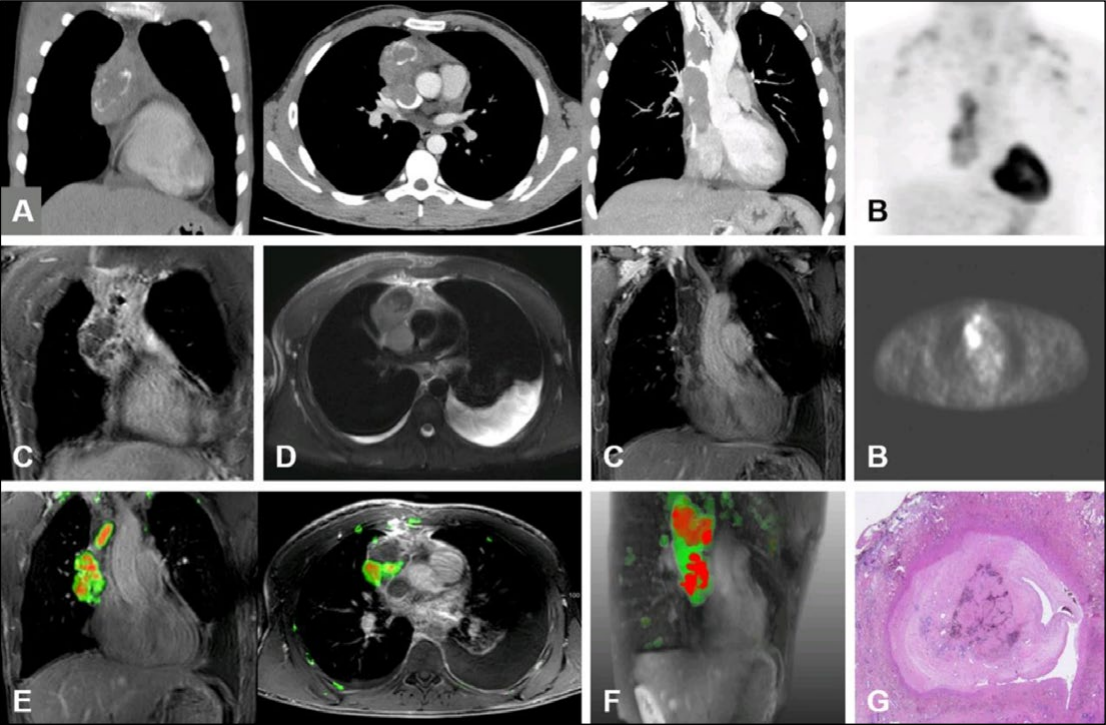
A 25-year-old man complains of hoarseness for several months. **(A and B)** PET-CT investigation showed a tumoral process in the anterior mediastinum with calcifications and thrombi in the VCS. The lesion and thrombus tissue are partially FDG avid. Fat-saturated **(C)** coronal and **(D)** axial T1-weighted MR images showed parallel results. **(E)** MPA maps in 2D and **(F)** 3D visualisations show that the malignant parts of this process are more centrally located, with invasion of the VCS. **(G)** The histopathological specimen with H&E stains confirmed thymus carcinoma with intravascular involvement. A laborious surgical resection with vascular reconstruction made a complete resection possible.

**Figure 4:**
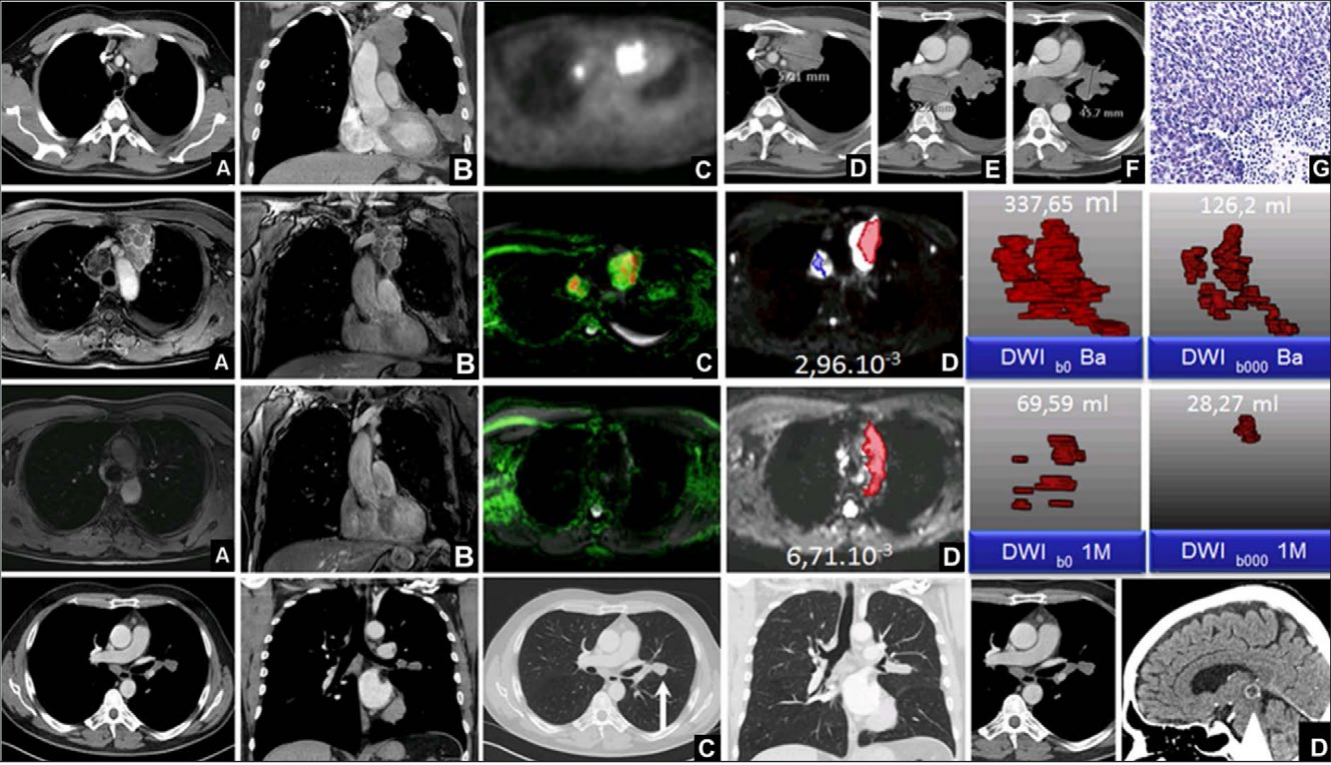
This collage of pictures is of a good CRT responder with SCLC-ED (PFS 236 days and OS 705 days). Upper row shows malignant cells in lymph nodes of region 4R and 6 on CT **(A and B)**, PET **(C)**, and in the left anterior mediastinum, 2R, 7 and 10L (*see spots **D, E, F***), which were histopathologically proven to be SCLC **(G)**. Second row shows axial, coronal MR Vibe **(A, B)**, with MPA **(C)** and b1000 image **(D)** at Base. The tumour volume load at b1000_Ba is 126.2 ml. The third row shows parallel images during therapy at 1M. TLV_b1000_1M is 28.27 ml. The fourth row shows an acceptable tumoral response after 6 months, but there is a new lesion left parahilar *(white arrow, **C**)* and after 24 months **(D)** a brain metastasis on the thalamus become visible *(arrowhead)*.

If these risk-zones were eccentrically located at the tumour edges or other morphological changes exist (e.g. lung infiltration, intravascular thrombus, presence of suspicious lymph nodes), these features dictate the surgical approach: only biopsy sampling before induction therapy, danger for incomplete resection, need for vascular reconstruction (Figure 3), or irresectability. Applying MPA the sensitivity, specificity and accuracy was 96,4% [54/56], 79,5% [35/44] and 89% [89/100], respectively. Finally, in a second phase we attempted to use DW-MR for early prediction of therapy outcome. Nowadays therapy response occurs by follow-up CT and PET-CT examinations using modified Response Evaluation Criteria in Solid Tumours (RECIST) criteria [8] (for MPM stage III and IV) or RECIST criteria [9] (for SCLC-ED). Anatomy-based assessments of treatment efficacy are, however, limited in their ability to predict outcome, which is partly due to possible delays in tumour load change after treatment. This kind of approach during early chemotherapy has already been published by many researchers [10,11]. The initial results of this study suggest that several DWI-volume and ADC-based parameters significantly correlate with overall survival and progression free survival [12]. This confirms the findings of Padhani et al. that DW-MR can act as a biomarker [13]. Notwithstanding there were some striking differences between these two cohorts. Patients with inoperable MPM were initially more resistant to chemotherapy if ADClow calculated from b values≤300 sec/mm² (was above 3 x 10^–3^ mm²/s), which we speculate indicating possibly a predominantly perfusion-related effect of chemotherapy. Patients suffering from SCLC-ED, which showed very good initial response, rather quickly developed distant metastases (Figure 4).

Although Webb WR published the first comprehensive review of MRI of the lung in 1985 [14], CT and PET are still currently the modalities applied for diagnosing and staging of chest diseases. The triptych publication of Biederer et al. [15,16,17] gives an overview of the possibilities with the MR modality. There are some reasons why MRI of the lung is still rarely used, except in a few centres, first is the lack of consistent protocols customised to clinical needs, second this technique is less accepted in the chest imaging community due to the phenomenon of multiple image artefacts, third many radiologists perceived MR imaging as too complicated: there are many contrast-influencing factors and parameters that affect image quality and last until now no sequence can deliver a lung image that has a resolution for exploring the second pulmonary lobule. Published data [18] suggest that effects of lung morphologic changes on functional parameters such as quantitative T2 mapping can provide additional information concerning the various interstitial lung disease (ILD) patterns and has the potential for monitoring of progression and response to treatment. Nevertheless, direct visualisation and morphological recognition of ILD features are the goal that must be achieved.

Since the group of Wild J. and Parra-Robles J. [19] presented an alternative and relatively less expensive method using natural-abundance polarized Xenon-129 gas, this research group developed a 3D alveolar capillary model that permits investigation of diffusion and perfusion coefficients measuring the ^129^Xe MR signal response [20]. Moreover, recently they compared ADC values obtained with ^129^Xe MR imaging with lobar ventilation in patients with chronic obstructive pulmonary disease (COPD) evaluated with quantitative CT metrics. They demonstrated good correlation between quantitative CT percentage emphysema on a lobar basis and ADC values obtained from ^129^Xe [21]. This opens horizons for diagnosis COPD patients who have benefit of regional treatments such as lung volume reduction surgery or endobronchial valve placement.

Also, the introduction of the hybrid PET-MR technique could be an opportunity, since staging plays an important role in oncology. Nowadays, PET-CT is the standard of care in staging lung cancer, combining the morphological information of CT with the metabolic information of PET. The novel hybrid PET-MR modality has the capability to provide structural imaging (morphological MR sequences), functional data (perfusion and diffusion), and molecular imaging (^18^FDG and other metabolisms), without the use of ionising radiation from a CT examination. The idea to characterize tissues based on different imaging features is not new. The concept is indeed very attractive, but standardisation of the different parameters leading to correct interpretation of tissue inhomogeneity remains difficult [22].

In this study it was found that the MRI examination at 3 Tesla can be carried out in safe conditions. We developed a conceptual approach for characterization of thoracic lesions, in diagnosis and differentiation (benign versus malignancy) in pleural, lung- and mediastinal lesions.
